# Radiotherapy and radiosurgery for benign skull base meningiomas

**DOI:** 10.1186/1748-717X-4-42

**Published:** 2009-10-14

**Authors:** Giuseppe Minniti, Maurizio Amichetti, Riccardo Maurizi Enrici

**Affiliations:** 1 Department of Radiotherapy Oncology, Sant' Andrea Hospital, University "La Sapienza", Rome, Italy; 2 Department of Neurosurgical Sciences, Neuromed Institute, Pozzilli (IS), Italy; 3 ATreP- Provincial Agency for Proton Therapy, Trento, Italy

## Abstract

Meningiomas located in the region of the base of skull are difficult to access. Complex combined surgical approaches are more likely to achieve complete tumor removal, but frequently at a cost of treatment related high morbidity. Local control following subtotal excision of benign meningiomas can be improved with conventional fractionated external beam radiation therapy with a reported 5-year progression-free survival up to 95%. New radiation techniques, including stereotactic radiosurgery (SRS), fractionated stereotactic radiotherapy (FSRT), and intensity-modulated radiotherapy (IMRT) have been developed as a more accurate technique of irradiation with more precise tumor localization, and consequently a reduction in the volume of normal brain irradiated to high radiation doses. SRS achieves a high tumour control rate in the range of 85-97% at 5 years, although it should be recommended only for tumors less than 3 cm away more than 3 mm from the optic pathway because of high risk of long-term neurological deficits. Fractionated RT delivered as FSRT, IMRT and protons is useful for larger and irregularly or complex-shaped skull base meningiomas close to critical structures not suitable for single-fraction SRS. The reported results indicate a high tumour control rate in the range of 85-100% at 5 years with a low risk of significant incidence of long-term toxicity. Because of the long natural history of benign meningiomas, larger series and longer follow-up are necessary to compare results and toxicity of different techniques.

## Introduction

Surgical excision is the treatment of choice for accessible intracranial meningiomas. Following apparently complete removal of benign meningiomas the reported control rates are in the region of 95% at 5 years, 90% at 10 years and 70% at 15 years [1-10]. However, meningiomas located in the region of the base of skull are often difficult to access and only subtotal or partial resection is possible, with a high tendency for tumor regrowth.

Local control following incomplete excision of a benign meningioma can be improved with conventional fractionated external beam radiotherapy (RT) with a reported 10-year progression-free survival in the region of 75-90% [11-13].

Advances in radiation oncology include intensity-modulated radiotherapy (IMRT), fractionated stereotactic radiotherapy (FSRT) and stereotactic radiosurgery (SRS) that allow for more localised and precise irradiation. Recent studies using these new techniques report apparently high local control rates and low morbidity for skull base benign tumors as pituitary adenomas, craniopharyngiomas and meningiomas [14-18]. We performed a review of the published literature of fractionated RT and SRS for skull base meningiomas in an attempt to define reasonably objective and comparative information on the safety and efficacy of the individual techniques.

## Conventional radiotherapy

Post-operative conventional RT has been reported effective both following subtotal surgical resection of benign meningiomas and at the time of recurrence. Using a dose of 50-55 Gy in 30-33 fractions the 10-year and 20-year local control rates are 70-80% (Table 1) [11-13,19-30].

**Table 1:** Summary of results on published studies on the conventional radiotherapy of skull base meningiomas

**authors**	**Patients** **(n)**	**S + RT** **(%)**	**RT** **(%)**	**Volume** **(ml)**	**Dose** **(Gy)**	**Follow-up** **(months)**	**Local control** **(%)**	**Late toxicity** **(%)**
Carella et al.,1982	57	84	16	NA	55 - 60	NA	95	NA
Forbes et al., 1984	31	100	0	NA	53	45	72 at 4 years	13
Barbaro et al., 1987	54	100	0	NA	52.5	78	68	0
Miralbell et al., 1992	36	100	0	NA	45-64	88	84 at 8 years	16
Goldsmith et al., 1994	117	100	0	NA	54	40	89 at 5 and 77 at 10 years	3.6
Maire et al., 1995	91	52	48	NA	52	40	94	6.5
Peele et al., 1996	42	100	0	NA	55	48	100	5
Condra et al.,1997	28	75	25	NA	53.3	98	87 at 15 years	24
Connell et al., 1999	54	80	20	NA	54	55	76 at 5 years	19
Maguire et al., 1999	26	78	22	NA	53	41	8 at 8 years	8
Nutting et al., 1999	82	100	0	NA	55-60	41	92 at 5 and 83 at 10 years	14
Vendrely et al., 1999	156	51	49	NA	50	40	79 at 5 years	11.5
Dufour et al., 2001	31	55	45	NA	52	73	93 at 5 and 10 years	3.2
Pourel et al., 2001	28	80	20	NA	56	30	95 at 5 years	4
Mendenhall et al., 2003	101	35	65	NA	54	64	95 at 5, 92 at 10 and 15 years	8

In a series of 82 patients with skull base meningiomas treated at the Royal Marsden Hospital between 1962 and 1992 using a dose of 55-60 Gy in 30-33 fractions, the 5-year and 10-year local tumor control rates were 92% and 83%, respectively [12]. Tumor site was the only significant predictor of local control, with a 10-year progression-free survival rate of 69% for patients with sphenoid ridge meningiomas as compared with 90% for those with tumors in the parasellar region. The overall 10-year survival rate for the entire cohort of patients was 71%, with performance status and patient age found to be significant independent prognostic factors. Goldsmith et al. [11] reported on 117 patients with benign meningiomas who were treated with conventional RT using a median dose of 54 Gy at University of California between 1967 and 1990. The 5-year and 10-year progression-free survival rates were 89% and 77%, and the respective survival 85% and 77%. A significant better progression-free survival was associated with a younger age and improvement of technologies. The 5-year progression-free survival rate for patients with benign meningioma treated after 1980 with three-dimensional (3-D) conformal RT was 98%, as compared with 77% for patients treated before 1980, with two-dimensional (2-D) RT. Similarly, Mendenhall et al [13] at a median follow-up of 5 years reported a local control in 101 patients treated with 3-D conformal RT of 95% at 5 years and 92% at 10 and 15 years, with a respective cause-specific survival rates of 97% and 92%, respectively. There were no difference between patients who underwent surgery and postoperative RT and patients who had RT alone. Overall, the actuarial 5-year and 10-year control rates reported in 5 publications (11-13,25,29) for a total of 359 patients were 90% (> 90% when a 3-D planning was used) and 83%, respectively.

Some tumor shrinkage after conventional RT has been reported in 10%-25% of patients. Local control after surgery implies complete removal of the tumor without evidence of regrowth on follow-up. In contrast, local control after radiation implies no evidence of progression on imaging. Benign tumors may regress partially, but they rarely disappear after successful irradiation. However, as long as there is no evidence of disease progression, the tumor is cured as effectively as thought it had been removed completely with surgery. The reported tumor control is similar for patients receiving a dose up to 60 Gy. Most of published series show no significant difference on tumor control with the use of doses ranging between 50 and 60 Gy, however a dose <50 Gy is associated with higher recurrence rates [13,22,28]. So far, in most centers, the standard dose for a benign meningioma is 55 Gy, whereas lower doses of 50-52 Gy are reserved for large meningiomas involving the optic pathways.

Analysis of treatment outcome after RT has lead to conflicting results. Size and tumor site have been reported as a predictor of tumor control. Connell et al [26] reported a 5-year control of 93% for 54 patients with skull base meningiomas less than 5 centimeters in greatest dimension and 40% for tumors more than 5 centimeters, and similar findings have been reported by others [11,23]. Nutting et al [12] found that patients with sphenoid ridge tumors had worse local control than other skull base meningiomas, and this was independent of the extent of surgery. Age and gender have not been a generally accepted prognostic factors for benign meningiomas, however younger age may be associated with better outcome in some series [11,12]. The reported local control and survival rates are similar for patients treated with RT as a part of their primary treatment or at the time of recurrence in most series [11-13,28]. However, only a prospective randomized trial can adequately determine whether the long-term control is influenced by timing of RT (early treatment versus delayed treatment after evidence of progression).

An important clinical endpoint of treatment is the improvement or the preservation of neurological function. Neurological deficits are usually present in up to 70% of patients with skull base meningiomas as consequence of tumor growth or previous surgery, and are mainly represented by deficits or II, III, IV, V and VI cranial nerves. An improvement or stabilization of neurological deficits are seen in up to 69% and 100% after conventional RT [12,13,23,28,29]. However most of the published series do not show any clinical result and clear figures about the functional outcome after conventional RT are lacking.

The toxicity of external beam RT is relatively low, ranging from 0 to 24% (Table 1), and includes the risk of developing neurological deficits, especially optic neuropathy, brain necrosis, cognitive deficits, and pituitary deficits. Cerebral necrosis with associated clinical neurological decline is a severe and sometimes fatal complication of RT, however remains exceptional when doses less than 60 Gy and 3-D planning system are used. Radiation injury to the optic apparatus may be manifest as decreased visual acuity or visual field defects and it is reported in 0-3% of irradiated patients with meningiomas. Amongst 82 patients with benign skull base meningiomas who were treated with conventional RT no cases of post-treatment optic nerve chiasm or other cranial nerve neuropathy were recorded [12]. Goldsmith et al [11] found a low incidence of radiation-induced optic neuropathy for dose less 55 Gy delivered to the optic pathways at conventional fractionation of 1,8-2 Gy per fraction. Parsons et al [31] observed no injuries in 106 optic nerves that received a total dose less than 59 Gy, whereas the 15-year actuarial risk of radiation-induced optic neuropathy was up to 47% in patients receiving a dose of 60 Gy or more using more than 1,9 Gy per fraction. Other cranial deficits are reported in less than 1-3% of patients. Hypopituitarism is reported in less than 5% of irradiated patients with skull base meningiomas, however hormone deficits are not systematically evaluated in the follow-up.

Certainly, patients with large parasellar meningiomas are at risk to develop late hypopituitarism and should be carefully assessed long-life after RT. Neurocognitive dysfunction is a recognized consequence of large volume RT for brain tumors [32] and has been occasionally reported in irradiated patients with meningiomas, especially impairment of short-term memory [23,27,29]. High dose radiation may be associated with the development of a second brain tumours. In a large series of 426 patients with pituitary adenomas who received conventional RT at the Royal Marsden Hospital between 1962 and 1994, the risk of second brain tumors was 2.0% at 10 yr and 2.4% at 20 yr, measured from the date of RT [33]. The relative risk of second brain tumor compared with the incidence in the normal population was 10.5 (95% CI, 4.3-16.7), being 7.0 for neuroepithelial and 24.3 for meningeal tumors.

In summary, conventional external beam radiation seems to be an efficient and safe initial or adjuvant treatment of benign meningiomas with a reported 10-year control rates more than 80% in most series, and compares favorably with tumor control rates reported after surgery alone, even after complete resection, suggesting that fractionated irradiation may produce at least a temporary tumor growth arrest. Neurological improvement has been reported in a significant number of patients with low toxicity in most cases.

## Fractionated stereotactic conformal radiotherapy (FSRT)

Assuming that fractionated RT is of value in achieving tumor control more sophisticated fractionated stereotactic radiation technique has been employed in patients with residual and recurrent meningiomas (Table 2) [34-43]. FSRT leads to a reduction in the volume of normal brain irradiated at high doses. Thus, the principal aim of radiosensitive structures sparing is to reduce the long-term toxicity of radiotherapy, and to increase the precision of treatment maintaining or possibly increasing its effectiveness.

**Table 2:** Summary of results on main published studies on the FSRT, IMRT, and proton radiotherapy of skull base meningiomas

**Authors**	**Technique**	**Patients** **(n)**	**S + SCRT** **(%)**	**SCRT** **(%)**	**Volume** **(ml)**	**Dose** **(Gy)**	**Follow-up** **(months)**	**Control rate** **(%)**	**Late toxicity** **(%)**
Debus et al., 2001	FSRT	189	69	31	52.5	56.8	35	97 at 5 and 96 at 10 years	12
Jalali et al., 2002	FSRT	41*	63	37	17.9	55	21	100	12.1
Lo et al., 2002	FSRT	18*	60	40	8.8	54	30.5	93.3	5
Torres et al., 2003	FSRT	77*	65	35	16.1	48.4	24	97.2	5.2
Selch et al., 2004	FSRT	45	64	36	14.5	56	36	100 at 3 years	0
Metellus et al., 2005	FSRT	38	20	18	12.7	53	88.6	94.7	2.6
Milker-Zabel et al., 2005	FSRT	317*	67	43	33.6	57.6	67	90.5 at 5 and 89 at 10 years	8.2
Henzel et al., 2006	FSRT	84	60	40	11.1	56	30	100	NA
Brell et al., 2006	FSRT	30	57	43	11.3	52	50	93 at 4 years	6.6
Hamm et al., 2008	FSRT	183*	70	30	27.4	56	36	97 at 5 years	8.2
Uy et al., 2002	IMRT	40*	62.5	27.5	20.2	50.4	30	93 at 5 years	5
Pirzkall et al., 2003	IMRT	20	80	20	108	57	36	100	0
Saja et al., 2005	IMRT	35*	54	46	NA	50.4	19.1	97 at 3 years	0
Milker-Zabel et al., 2007	IMRT	94**	72	28	81.4	57.6	52	93.6	4
Wenkel et al, 2000	Ph + protons	46*	83	17	76	59	53	100 at 5 and 88 at 10 years	16
Vernimmen et al, 2001	protons	23*	65	35	23.3°	20,6*	38°	87	13
Weber et al., 2004	protons	16*	81	19	17.5	56	34.1	91.7 at 3 years	24
Noel et al, 2005	Ph + protons	51*	86	14	17	60.6	21	98 at 4 years	4

S, surgery; FSRT, stereotactic conformal radiotherapy; IMRT, intensive modulated radiotherapy; Ph, photons

*series includes some intracranial meningiomas

**series includes some atypical/malignant meningiomas

° mean

In a series of 41 patients with benign residual or recurrent meningiomas treated at the Royal Marsden Hospital with FSRT between 1994 and 1999 [35] at a median follow-up of 21 months (range 2-62 months) none of patients have recurred. Using a dose of 55 Gy in 33 fractions the actuarial survival rates were 100% at 2 years and 91% at 3 and 5 years. Tumor control was similar between patients treated post-operatively and patients treated with FSRT alone, regardless the sex, age, tumor site and irradiated volume. Debus et al [34] reported on 189 patients with large benign skull base meningiomas treated with FSRT with a mean radiation dose of 56.8 Gy at University of Heidelberg. At a median follow-up of 35 months (range, 3 months to 12 years) they reported a 5-year tumor control and survival of 94% and 97%, respectively. A volume reduction of more than 50% was observed in 14% of patients. A recent up-date of 317 patients treated at the same Institution showed, at a median follow-up of 5.7 years, a 5-year and 10-year tumor control of 90.5% and 89%, and respective survival of 95% and 90% [40]. Patients treated for recurrent meningioma showed a trend toward decreased progression-free survival compared with patients treated with primary therapy after subtotal resection. Patients with a tumor volume more than 60 cm^3 ^had a significant recurrence rate of 15.5% vs. 4.3% for those with a tumor volume of 60 cm^3 ^or less (p < 0.001). Hamm et al [43] at a median follow-up of 36 months reported a 5-year tumor control and survival of 93% and 97% in 183 patients with large skull base meningiomas. A partial imaging response occurred in 23% of patients, and in the 95% of patients the neurological symptoms improved or remained stable. In a series of 27 patients with large recurrent benign skull base meningiomas (> 4 cm) treated at our Institution with FSRT between 2005 and 2009 at a median dose of 50 Gy in 30 daily fractions the 2-year local control and survival were 100% [44]. Eight patients (29%) showed a tumor shrinkage more than 25% during the follow-up. Although majority of the tumors treated had irregular shape and compressed the optic chiasm, no visual deficits have been recorded during the follow-up.

A clinical neurological improvement is reported in 14-44% of patients after FSRT [34,39,41,43]. A late significant toxicity is reported in less than 5% of patients, including cranial deficits (leading especially to visual problems), hypopituarism and impairment for neurocognitive function (Table 2). However, the evaluation of complications is often subjective and unsatisfactory, so that well designed prospective studies are needed to better evaluate the true incidence of long-term side effects comparing the different techniques. No cases of second tumor after FSRT for meningiomas have been reported to date. On theoretical grounds, the reduction of the volume of normal brain receiving high radiation doses using FSRT may decrease the risk of radiation-induced tumors, however to demonstrate a change in the incidence of second brain tumors will require large series of patients with appropriate follow-up of 10-20 years.

In summary, FSRT is an effective and safe treatment modality for local control of skull base meningiomas and tumor control is comparable to the reported results of other fractionated radiation techniques and SRS for benign skull base meningiomas. FSRT offers a more localized irradiation compared with conventional RT and the reported data from literature indicate that radiation induced morbidity is quite low. Although longer follow-up is necessary to clearly demonstrate the potential reduction of long term complications in comparison with conventional RT, currently FSRT should be preferred for the radiation treatment of large skull base tumors, especially those in close proximity to the optic apparatus.

## Intensity modulated radiotherapy (IMRT)

IMRT represents an advanced form of 3-D conformal which has been recently employed for the treatment of different brain tumors, especially large tumors with irregular shapes close to critical structures [45]. IMRT for meningiomas results in a more conformality and better target coverage than CRT and therefore able to spare more radiosensitive brain structures [46]. IMRT uses a series of multiple subfields created by MLC which move under computer control creating modulated fields. IMRT treatment plans are generated using inverse planning system, which uses computer optimization techniques to modulate intensities across the target volume and sensitive normal structures, starting from a specified dose distribution.

Few series are available on the use of IMRT in patients with meningiomas (Table 2) [16,47-49]. Milker-Zabel et al [16] reported on 94 patients with complex-shaped meningiomas treated with IMRT at University of Heidemberg between 1998 and 2004. At a median follow-up of 4.4 years, the reported tumor local control was 93.6%. Recurrence-free survival in patients with WHO Grade 1 meningiomas was 97.5% at 3 years and 93.6% at 5 years, and overall survival was 97%. Sixty-nine patients had stable disease based on CT/MRI, whereas 19 had a tumor volume reduction, and 6 patients showed tumor progression after IMRT. A neurological improvement was noted in about 40% of patients and a worsening of preexisting neurologic symptoms was seen in 4% of patients. No secondary malignancies were seen after IMRT, however this may simply be a reflection of the lack of adequate long-term follow-up. Similar results have been reported by others in some small series, with a reported local control of 93-97% at median follow-up of 19-36 months and low toxicity [47-49], suggesting that IMRT is a feasible treatment modality for control of complex-shaped meningioma. In summary, IMRT allows the delivery of a high dose to such complex-shaped skull base tumors while sparing the surrounding radiosensitive structures, especially optic chiasm and brainstem, although longer follow-up does needs to confirm the potential reduction of radiation-induced toxicity of IMRT in comparison with 3D conformal RT in large skull base meningiomas.

## Proton radiotherapy

Proton irradiation can achieve better target-dose conformality when compared to 3D-CRT and IMRT and the advantage becomes more apparent for large volumes. Distribution of low and intermediate doses to portions of irradiated brain are significant lower with protons when compared with photons and also could favor the use of protons in younger patients Moreover, proton therapy can be delivered as stereotactic radiosurgery or as fractionated stereotactic radiotherapy with the same used immobilization systems and target accuracy of photon techniques.

Tumor control after proton beam RT is shown in Table 2[50-53]. Noel et al [53] reported on 51 patients with skull base meningiomas treated between 1994 and 2002 with a combination of photon and proton RT at Institute Curie in Orsay. At a median follow-up of 25.4 months the 4-year local control and overall survival rates were 98% and 100%, respectively. Neurological improvement was reported in 69% of patients and stabilization in 31%. Wenkel et al [50] reported on 46 patients with partially resected or recurrent meningiomas treated between 198 and 1996 with combined photon and proton beam therapy at the Massachusetts General Hospital (MGH). At a median follow-up of 53 months overall survival at 5 and 10 years was 93 and 77%, respectively, and the recurrence-free rate at 5 and 10 years was 100% and 88%, respectively. Three patients had local tumor recurrence at 61, 95, and 125 months. Seventeen percent of patients developed severe long-term toxicity from RT, including ophthalmologic, neurologic, and otologic complications. At a median follow-up of 40 months a tumor control of 89% has been reported by Vernimmen et al [51] in in 27 patients with large skull base meningiomas (median volume 43.7 cm^3^) treated with stereotactic proton beam therapy. Permanent neurological deficits were reported in 3 patients.

In summary, proton irradiation alone or in combination with photons is effective in controlling meningiomas, with a tumor control and toxicity in the range of photon therapy. On the basis of the dosimetric advantages of protons, including better conformality and reduction of integral radiation dose to normal tissue, fractionated proton irradiation may be considered in patients with large and/or complex-shaped meningiomas or younger patients, possibly limiting the long-term late effects of irradiation. As more hospital-based proton treatment centers are becoming operational, prospective trials that assess the late toxicity of different radiation techniques are needed to confirm the expected reduction in long-term side effects with proton RT.

## Stereotactic radiosurgery (SRS)

Since 1990, either Gamma Knife (GK) or Linear Accelerator (LINAC) have been extensively employed in the radiosurgical treatment of skull base meningiomas. A summary of main recent published series of SRS in skull base meningiomas is shown in Table 3[36,37,39,54-84]. Differing from the earliest reports with short follow-ups, large recently published series report a more appropriate 5-year and 10-year actuarial control rates. In a large series of 972 patients mostly with skull base meningiomas, who underwent Gamma GK SRS at the University of Pittsburgh, the reported actuarial tumor control rates were 93% at 5 years and 87 at 10 and 15 years using a median dose to the tumor margin of 13 Gy, with no differences between 384 patients who underwent postoperative SRS and 488 patients treated with primary SRS [18]. These result confirm a previous study of 159 patients treated with GK SRS at the same Institution with a reported actuarial tumor control rate for patients with typical meningiomas of about 93% at both 5 and 10 years [70]. Tumor volumes decreased in 3%, remained stable in 60%, and increased in 6% of patients. Kreil et al [75] in 200 patients with skull base meningiomas treated with GK SRS reported a 5-year and 10-year local control of 98.5% and 97%, respectively, and similar results have been reported in some recent large series including more than 100 patients [67,69,70,72,75,77,78,81,83]. Overall, eighteen studies including 2919 skull base meningiomas report a 5-year actuarial control of 91%; amongst them, 7 studies including 1626 skull base meningiomas report a 10-year actuarial control of 87.6% (Table 3). Although in most series radiosurgical dose has been delivered using GK SRS, a similar outcome has been reported with the use of LINAC SRS.

**Table 3:** Summary of results on main published studies on the radiosurgery of skull base meningiomas

**authors**	**patients**	**type**	**S + RS** **%**	**RS** **%**	**tumor volume** **median (ml)**	**median dose** **Gy**	**follow-up** **median (months)**	**local control** **%**	**volume** **reduction (%)**	**neurological** **improvement**	**Toxicity** **%**
valentino et al., 1993	72	LINAC	53	47	NA	NA	44	93	69	50	6.7
Hudgins et al., 1996	100*	GK	91	9	14	15	NA	91	47	8	12
Kurita et al., 1997	18	GK	83	17	NA	17	34.8	87.5 at 5 years	35	NA	49.9
Chang et al., 1998	24	GK	66	34	6.8	17.7	45.6	100	37	42	33
Pan et al., 1998	63	GK	54	46	NA	NA	21	91	74	37.5	7.5
Morita et al., 1999	88	GK	55	45	8.1	16	35	95 at 5 years	70	NA	14.8
Shafron et al., 1999	50	LINAC	46	54	10	12.7	23	100	44	NA	3
Liscak et al., (1999)	67	GK	36	64	7.8	12	19	100	52	35.8	3.8
Aichholzer et al., 2000	46*	GK	67	33	NA	15.9	48	97.5	52	33	13.2
Roche et al., 2000	80	GK	37	63	4.7	14	30.5	92.8 at 5 years	31	43	5
Villavicencio et al., 2001	56	LINAC	64	36	6	15	26	95	44	34	9
Kobayashi et al., 2001	87	GK	56	44	NA	14	30°	89 at 7 years	23	48	13.8
Shin et al., 2001	40	GK	66	34	4.3	18	40	82.3 a 10 years	37	20	22.5
Stafford et al., 2001	190*	GK	59	41	8.2	16	47	93 at 5 years	56	8	13
Spiegelmann et al., 2002	42	LINAC	26	74	8.4	14	36	97.5 at 7 years	60	22	22.4
Nicolato et al., 2002	111	GK	49	51	10	14.8	48.2	96 at 5 years	63	66	8
Lee et al., 2002	155	GK	46	54	6.5	15	35	93 at 5 and 10 years	34	29	6.7
Lo et al., 2002	35	LINAC	60	40	6.8	14	38	92.7 at 3 years	37.5	NA	6
Eustachio et al., 2002)	121	GK	49	51	6.8	13	82	97.8	60	44	6.7
Torres et al., 2003	77*	LINAC	65	35	12.7	15.6	40.6	92.1	35	35	5
DiBiase et al., 2004	162	GK	38	62	4.5	14	54	86.2 at 5 years	28	NA	8.3
Deinsberger et al., 2004	37	LINAC	22	78	5.9	14.6	66	97.2	32	NA	5.6
Pollock et al., 2005	49	GK	0	100	10.2	16	58	85 at 3 and 80 at 7 years	59	26	20
Kreil et al., 2005	200	GK	50.5	49.5	6.5	12	95	98.5 at 5 and 97 at 10 years	56.5	41	4.5
Zachenhofer et al., 2006	36	GK	70	30	NA	17	103	94	53	36	5
Kollova et al., 2007	368	GK	30	70	4.4	12.5	60	98 at 5 years	69	62	15.9
Hasewaga et al., 2007	115	GK	57	43	14	13	62	87 at 5 and 73 at 10 years	51	46	12
Feigl et al., 2007	214	GK	43	57	6.5°	13.6°	24°	86.3 at 4 years	74	19	6.7
Davidson et al., 2007	36	GK	100	0	4.1	16	81	100 at 5 and 94.7 at 10 years	14	44	3
Kondziolka et al., 2008	972*	GK	49	51	7.4*	14°	48°	87 at 10 and 15 years	42	35	7.7
Iway et al., 2008	108	GK	NA	NA	8.1	12	86.1	93 at 5 and 83 at 10 years	46	21	6
Han et al., 2008	98	GK	36	64	6.3°	12.7	77°	90 at 5 years	44	45	16
Takanashi et al., 2009	101	GK	24	76	7.1	13.2	52°	97%	44	45	0
Ganz et al., 2009	97	GK	NA	NA	15.9°	12°	53°	100% at 2 years	31	NA	3

*series include skull base and intracranial meningiomas;

°mean

Only few studies have compared the outcome of SRS and FSRT in skull base meningiomas [36,37,39]. Metellus et al [39] found no differences in tumor control between 38 patients treated with fractionated RT and 36 patients treated with SRS. Actuarial progression-free survival was 94.7% in fractionated RT group and 94.4% in SRS group, with permanent morbidity of 2.6% after FSRT and 0% after SRS. Torres et al [37] reported on 77 patients treated with SRS and 51 patients treated with FSRT. Tumor control was achieved in 90% of patients at a median follow-up of 40 months after SRS, and in 97% of patients at a median follow-up of 24 months following FSRT. Late complications were recorded in 5% of patients treated with SRS and 5.2% patients treated with FSRT. A similar 3-year local control of 94% has been reported by Lo et al [36] in 35 patients treated with SRS and in 18 patients with large tumors treated with FSRT. Permanent morbidity was 2.6% in SRS group and 0% in FSRT group. These data suggests that either SRS or FSRT are safe and effective techniques in the treatment of skull base meningiomas, affording comparable satisfactory long-term tumor control. The main differences between FSRT group and SRS group treated at the same Institution was the average diameter of meningiomas or the close proximity to sensitive structures. Patients with tumors less than 3 cm and more than 3-5 mm away from radiosensitive structures, such as optic chiasm or brainstem, were selected for SRS whereas FSRT was employed for all tumors that were not amenable to SRS. In our Institution both stereotactic techniques are available and the we recommend FSRT for skull base tumors that are - more than 3 cm; - in close proximity of the optic chiasm (less than 3-5 mm); compressing the brainstem and - with irregular margins.

Radiosurgical doses between 12 and 18 Gy have been used in the control of skull base meningiomas. Over the last years, SRS doses have been decreased with the aim to minimize long-term toxicity while maintaining efficacy. Ganz et al [84] reported on 97 patients with meningiomas with median volume of 15.9 cm^3 ^treated with GK SRS using a dose of 12 Gy. At A median follow-up of 54 months the 2-year progression-free survival was 100%. Twenty-seven were smaller and 72 unchanged in volume. Three patients suffered adverse radiation effects. Overall, at median dose of 12-14 Gy the reported 5-year actuarial tumor control rate remains in the range of 90-95% as for higher doses [74,77,79-84].

The rate of tumor shrinkage measured varied in all studies, ranging from 16% to 69% in the different series, and tends to increase in patients with longer follow-up. Similarly, a variable improvement of neurological functions has been shown in 10-60% of patients, however the evaluation of neurological improvement is frequently retrospective and the criteria used to evaluate the functional improvement are subjective or not available in most series.

Analysis of factors predicting local tumor control in most series shows no significant differences between patients underwent SRS as primary treatment and patients treated for incomplete resected or recurrent meningioma. Age, sex, site of meningioma, and neurological status did not affect significantly the outcome in most published series, however larger meningiomas are associated with worse long-term local control [18,72]. DiBiase et al [72] reported a significant higher 5-year tumor control in patients with meningiomas < 10 ml than those with larger tumors (92% vs 68%, p = 0.038). In a recent series of 972 patients with meningioma poorer local control was correlated with increasing volume (p = 0.01), and a similar trend was observed with disease-specific survival (p = 0.11) [18].

More recently the image-guided robotic radiosurgery system (Cyberknife) has been employed for frameless SRS in patients with skull base meningiomas [85,86]. Patient position and motion are measured by two diagnostic x-ray cameras and communicated in real time to the robotic arm for beam targeting and patient motion tracking. Although patients are fixed in a thermoplastic mask, the system achieves the same level of targeting precision as conventional frame-based RS. Colombo et al [86] in a series of 199 benign intracranial meningiomas (157 skull base meningiomas) reported a 5-year control of 93.5%. Tumors larger than 8 ml and/or situated close to critical structures were treated with hypofractionated stereotactic RT (2 to 5 daily fractions). The tumor volume decreased in 36 patients, was unchanged in 148 patients, and increased in 7 patients. Clinical symptoms improved in 30 patients. Tumor control in 63 patients with tumor volume up 65 ml treated with hypofractionated RT was similar to that obtained in smaller meningiomas treated with single fraction SRS. Neurological deterioration was observed in 4% of patients, represented mainly by visual deficits. Although the small numbers of fractions possible with the CyberKnife seems safer than SRS for large parasellar meningiomas, further large series with appropriate follow-up should confirm the low risk of optic neuropathy in patients treated with hypofractionated regimens. Currently for large meningiomas close to the optic pathways, in our opinion FSRT should be chosen based on its proven efficacy and safety.

Complications of SRS are reported in 3 to 40% of cases (corrected mean 8%), being represented by either transient (3.0%) or permanent complications (5.0%). Although radionecrosis of the brain and delayed cranial nerve deficits after SRS are of concern, the rate of significant complications at doses of 12-15 Gy as currently used in most centers is less than 6% (Table 3). Kondziolka et al [18] reported a permanent neurological deficits of 9% at 10 and 15 years in 972 patients treated with GK SRS for intracranial meningiomas. The morbidity rate for cavernous sinus meningiomas was 6.3%, including visual deterioration, 6^th ^nerve palsy, and trigeminal neuropathy. In the series of Nicolato et al [69] late complications occurred in 4.5% of patients, being transient in 80% of them, and similar complication rates have been reported in all main published series (Table 3). Few cases of radiation induced tumors, mainly glioblastoma, have been reported in the literature [84,87-91], however the real incidence of second brain tumors cannot be clearly established because of short follow-up reported in the majority of radiosurgical series. Other complications, as epilepsy, internal carotid occlusion, and hypopituitarism have been rarely reported (less than 1-2%).

The risk of clinically significant radiation optic neuropathy for patients receiving SRS for skull base meningiomas is 1-2% following doses to optic chiasm below 10 Gy and this percentage may significantly increase for higher doses [56,85,86]. Leber et al [92] reviewed 50 patients having SRS for benign skull base tumors in which the optic nerves or chiasm were exposed to 4.5 Gy or more. For patients receiving 10 to 15 Gy and greater than 15 Gy, the risk of radiation-induced optic neuropathy was 26.7% and 77.8%, respectively, however no optic neuropathy was observed when a dose less than 10 Gy was delivered to the optic apparatus. Stafford et al [93] found that the risk of developing a clinically significant optic neuropathy was 1.1% for patients receiving a point maximum dose of 12 Gy or less, and similar results have been reported by others [59]. Considering an effective dose of 13-16 Gy to achieve local control of a skull base meningioma and a recommended dose of 8 Gy as the maximum for the optic chiasm, in clinical practice this means that a distance between tumor margin and optic apparatus should be at least of 2-3 mm to avoid visual deterioration. In contrast motor cranial nerve deficits in the cavernous sinus rarely have been reported with doses less than 16 Gy. For meningiomas involving the clivus and cerebellopontine angle the estimated tolerance dose for the brainstem is 15 Gy, however facial nerve and acoustic injuries may occur at lower doses.

In summary, SRS may represents a convenient and safe approach for patients with skull base meningiomas with a tumor control at 5 and 10 years comparable to fractionated RT. Both SRS and FSRT are effective treatment options for benign skull base meningiomas and the choice of stereotactic technique is mainly based on the characteristics of tumors. In most centers SRS is usually reserved for tumors less than 3 cm away 3-5 mm from the optic chiasm, whereas FSRT is employed for those tumors not amenable to SRS. The reported toxicity of SRS is low when doses of 13-15 Gy are used. Although the risk of a second tumor after SRS is of concern, the reported low incidence should not preclude the use SRS as an effective treatment modality in patients with skull base meningioma.

## Conclusion

Radiation is highly effective in the management of skull base meningioma and long-term data clearly indicate a tumor control in more than 80% of patients after 10 years, with an acceptable incidence of complications. Stereotactic techniques (RS and FSRT) offer a more localized irradiation compared with conventional radiotherapy and has the potential of reducing the risk of long term radiation induced morbidity. Currently SRS and FSRT represent the commonest treatment modality of irradiation for skull base meningiomas, providing a comparable high rates of long-term tumor control with low morbidity. The choice of stereotactic technique should be based on tumor characteristics. SRS is usually suitable only in selected patients, whereas there is no restriction to the size and the position meningioma suitable for standard dose fractionated radiotherapy. Current practice aims to avoid irradiating the optic apparatus beyond single doses of 8-10 Gy. This means that RS is usually offered to patients with relatively small skull base meningiomas not in close proximity of optic apparatus. Hypofractionated stereotactic RT in patients with large skull base meningiomas abutting the optic pathway is a promising treatment, however more robust data need to definitively evaluate the long-term efficacy and toxicity of hypofractionation. Proton irradiation may be considered in patients with large and complex-shaped meningiomas or younger patients, with the aim to limit the long-term late effects of irradiation. Because of slow-growing potential of meningiomas, the superiority of the individual techniques need to be confirmed in prospective and methodologically rigorous studies with appropriate 10-20 years follow-up.

## Competing interests

The authors declare that they have no competing interests.

## Authors' contributions

GM conceived and drafted the manuscript. MA helped the draft and participated in its design. RME critically reviewed/revised the article. All authors read and approved the final manuscript.

## References

[B1] Sekhar LN, Jannetta PJ (1984). Cerebellopontine angle meningiomas. J Neurosurg.

[B2] Samii M, Ammirati M, Mahran A, Bini W, Sepehrnia A (1989). Surgery of petroclival meningiomas: report of 24 cases. Neurosurgery.

[B3] Kallio M, Sankila R, Hakulinen T, Jääskeläinen J (1992). Factors affecting operative and excess long-term mortality in 935 patients with intracranial meningioma. Neurosurgery.

[B4] DeMonte F, Smith HK, al-Mefty O (1994). Outcome of aggressive removal of cavernous sinus meningiomas. J Neurosurg.

[B5] Cusimano MD, Sekhar LN, Sen CN, Pomonis S, Wright DC, Biglan AW, Jannetta PJ (1995). The results of surgery for benign tumors of the cavernous sinus. Neurosurgery.

[B6] De Jesús O, Sekhar LN, Parikh HK, Wright DC, Wagner DP (1996). Long-term follow-up of patients with meningiomas involving the cavernous sinus: recurrence, progression, and quality of life. Neurosurgery.

[B7] O'Sullivan MG, van Loveren HR, Tew JM (1997). The surgical resectability of meningiomas of the cavernous sinus. Neurosurgery.

[B8] Abdel-Aziz KM, Froelich SC, Dagnew E, Jean W, Breneman JC, Zuccarello M, van Loveren HR, Tew JM (2004). Large sphenoid wing meningiomas involving the cavernous sinus: conservative surgical strategies for better functional outcomes. Neurosurgery.

[B9] Shrivastava RK, Sen C, Costantino PD, Della Rocca R (2005). Sphenoorbital meningiomas: surgical limitations and lessons learned in their long-term management. J Neurosurg.

[B10] Sindou M, Wydh E, Jouanneau E, Nebbal M, Lieutaud T (2007). Long-term follow-up of meningiomas of the cavernous sinus after surgical treatment alone. J Neurosurg.

[B11] Goldsmith BJ, Wara WM, Wilson CB, Larson DA (1994). Postoperative irradiation for subtotally resected meningiomas. J Neurosurg.

[B12] Nutting C, Brada M, Brazil L, Sibtain A, Saran F, Westbury C, Moore A, Thomas DG, Traish D, Ashley S (1999). Radiotherapy in the treatment of benign meningioma of the skull base. J Neurosurg.

[B13] Mendenhall WM, Morris CG, Amdur RJ, Foote KD, Friedman WA (2003). Radiotherapy alone or after subtotal resection for benign skull base meningiomas. Cancer.

[B14] Brada M, Ajithkumar TV, Minniti G (2004). Radiosurgery for pituitary adenomas. Clin Endocrinol (Oxf).

[B15] Minniti G, Traish D, Ashley S, Gonsalves A, Brada M (2006). Fractionated stereotactic conformal radiotherapy for secreting and nonsecreting pituitary adenomas. Clin Endocrinol (Oxf).

[B16] Milker-Zabel S, Zabel-du Bois A, Huber P, Schlegel W, Debus J (2007). Intensity-modulated radiotherapy for complex-shaped meningioma of the skull base: long-term experience of a single institution. Int J Radiat Oncol Biol Phys.

[B17] Minniti G, Saran F, Traish D, Soomal R, Sardell S, Gonsalves A, Ashley S, Warrington J, Burke K, Mosleh-Shirazi A, Brada M (2007). Fractionated stereotactic conformal radiotherapy following conservative surgery in the control of craniopharyngiomas. Radiother Oncol.

[B18] Kondziolka D, Mathieu D, Lunsford LD, Martin JJ, Madhok R, Niranjan A, Flickinger JC (2008). Radiosurgery as definitive management of intracranial meningiomas. Neurosurgery.

[B19] Carella RJ, Ransohoff J, Newall J (1982). Role of radiation therapy in the management of meningioma. Neurosurgery.

[B20] Forbes AR, Goldberg ID (1984). Radiation therapy in the treatment of meningioma: the Joint Center for Radiation Therapy experience 1970 to 1982. J Clin Oncol.

[B21] Barbaro NM, Gutin PH, Wilson CB, Sheline GE, Boldrey EB, Wara WM (1987). Radiation therapy in the treatment of partially resected meningiomas. Neurosurgery.

[B22] Miralbell R, Linggood RM, de la Monte S, Convery K, Munzenrider JE, Mirimanoff RO (1992). The role of radiotherapy in the treatment of subtotally resected benign meningiomas. J Neurooncol.

[B23] Maire JP, Caudry M, Guérin J, Célérier D, San Galli F, Causse N, Trouette R, Dautheribes M (1995). Fractionated radiation therapy in the treatment of intracranial meningiomas: local control, functional efficacy, and tolerance in 91 patients. Int J Radiat Oncol Biol Phys.

[B24] Peele KA, Kennerdell JS, Maroon JC, Kalnicki S, Kazim M, Gardner T, Malton M, Goodglick T, Rosen C (1996). The role of postoperative irradiation in the management of sphenoid wing meningiomas. Ophthalmology.

[B25] Condra KS, Buatti JM, Mendenhall WM, Friedman WA, Marcus RB, Rhoton AL (1997). Benign meningiomas: primary treatment selection affects survival. Int J Radiat Oncol Biol Phys.

[B26] Connell PP, Macdonald RL, Mansur DB, Nicholas MK, Mundt AJ (1999). Tumor size predicts control of benign meningiomas treated with radiotherapy. Neurosurgery.

[B27] Maguire PD, Clough R, Friedman AH, Halperin EC (1999). Fractionated external-beam radiation therapy for meningiomas of the cavernous sinus. Int J Radiat Oncol Biol Phys.

[B28] Vendrely V, Maire JP, Darrouzet V, Bonichon N, San Galli F, Célérier D, Causse N, Demeaux H, Trouette R, Dahan O, Récaldini L, Guérin J, Caudry M (1999). [Fractionated radiotherapy of intracranial meningiomas: 15 years' experience at the Bordeaux University Hospital Center]. Cancer Radiother.

[B29] Dufour H, Muracciole X, Métellus P, Régis J, Chinot O, Grisoli F (2001). Long-term tumor control and functional outcome in patients with cavernous sinus meningiomas treated by radiotherapy with or without previous surgery: is there an alternative to aggressive tumor removal?. Neurosurgery.

[B30] Pourel N, Auque J, Bracard S, Hoffstetter S, Luporsi E, Vignaud JM, Bey P (2001). Efficacy of external fractionated radiation therapy in the treatment of meningiomas: a 20-year experience. Radiother Oncol.

[B31] Parsons JT, Bova FJ, Fitzgerald CR, Mendenhall WM, Million RR (1994). Radiation optic neuropathy after megavoltage external-beam irradiation: analysis of time-dose factors. Int J Radiat Oncol Biol Phys.

[B32] Crossen JR, Garwood D, Glatstein E, Neuwelt EA (1994). Neurobehavioral sequelae of cranial irradiation in adults: a review of radiation-induced encephalopathy. J Clin Oncol.

[B33] Minniti G, Traish D, Ashley S, Gonsalves A, Brada M (2005). Risk of second brain tumor after conservative surgery and radiotherapy for pituitary adenoma: update after an additional 10 years. J Clin Endocrinol Metab.

[B34] Debus J, Wuendrich M, Pirzkall A, Hoess A, Schlegel W, Zuna I, Engenhart-Cabillic R, Wannenmacher M (2001). High efficacy of fractionated stereotactic radiotherapy of large base-of-skull meningiomas: long-term results. J Clin Oncol.

[B35] Jalali R, Loughrey C, Baumert B, Perks J, Warrington AP, Traish D, Ashley S, Brada M (2002). High precision focused irradiation in the form of fractionated stereotactic conformal radiotherapy (SCRT) for benign meningiomas predominantly in the skull base location. Clin Oncol (R Coll Radiol).

[B36] Lo SS, Cho KH, Hall WA, Kossow RJ, Hernandez WL, McCollow KK, Gerbi BJ, Higgins PD, Lee CK, Dusenbery KE (2002). Single dose versus fractionated stereotactic radiotherapy for meningiomas. Can J Neurol Sci.

[B37] Torres RC, Frighetto L, De Salles AA, Goss B, Medin P, Solberg T, Ford JM, Selch M (2003). Radiosurgery and stereotactic radiotherapy for intracranial meningiomas. Neurosurg Focus.

[B38] Selch MT, Ahn E, Laskari A, Lee SP, Agazaryan N, Solberg TD, Cabatan-Awang C, Frighetto L, Desalles AA (2004). Stereotactic radiotherapy for treatment of cavernous sinus meningiomas. Int J Radiat Oncol Biol Phys.

[B39] Metellus P, Regis J, Muracciole X, Fuentes S, Dufour H, Nanni I, Chinot O, Martin PM, Grisoli F (2005). Evaluation of fractionated radiotherapy and gamma knife radiosurgery in cavernous sinus meningiomas: treatment strategy. Neurosurgery.

[B40] Milker-Zabel S, Zabel A, Schulz-Ertner D, Schlegel W, Wannenmacher M, Debus J (2005). Fractionated stereotactic radiotherapy in patients with benign or atypical intracranial meningioma: long-term experience and prognostic factors. Int J Radiat Oncol Biol Phys.

[B41] Brell M, Villà S, Teixidor P, Lucas A, Ferrán E, Marín S, Acebes JJ (2006). Fractionated stereotactic radiotherapy in the treatment of exclusive cavernous sinus meningioma: functional outcome, local control, and tolerance. Surg Neurol.

[B42] Henzel M, Gross MW, Hamm K, Surber G, Kleinert G, Failing T, Strassman G, Engenhart-Cabillic R (2006). Significant tumor volume reduction of meningiomas after stereotactic radiotherapy: results of a prospective multicenter study. Neurosurgery.

[B43] Hamm K, Henzel M, Gross MW, Surber G, Kleinert G, Engenhart-Cabillic R (2008). Radiosurgery/stereotactic radiotherapy in the therapeutical concept for skull base meningiomas. Zentralbl Neurochir.

[B44] Minniti G, Muni R (2008). Radiotherapy for sellar and parasellar tumors. Rivista Medica.

[B45] Intensity Modulated Radiation Therapy Collaborative Working Group Intensity-modulated radiotherapy (2001). current status and issues of interest. Int J Radiat Oncol Biol Phys.

[B46] Pirzkall A, Carol M, Lohr F, Höss A, Wannenmacher M, Debus J (2000). Comparison of intensity-modulated radiotherapy with conventional conformal radiotherapy for complex-shaped tumors. Int J Radiat Oncol Biol Phys.

[B47] Uy NW, Woo SY, Teh BS, Mai WY, Carpenter LS, Chiu JK, Lu HH, Gildenberg P, Trask T, Grant WH, Butler EB (2002). Intensity-modulated radiation therapy (IMRT) for meningioma. Int J Radiat Oncol Biol Phys.

[B48] Pirzkall A, Debus J, Haering P, Rhein B, Grosser KH, Höss A, Wannenmacher M (2003). Intensity modulated radiotherapy (IMRT) for recurrent, residual, or untreated skull-base meningiomas: preliminary clinical experience. Int J Radiat Oncol Biol Phys.

[B49] Sajja R, Barnett GH, Lee SY, Harnisch G, Stevens GH, Lee J, Suh JH (2005). Intensity-modulated radiation therapy (IMRT) for newly diagnosed and recurrent intracranial meningiomas: preliminary results. Technol Cancer Res Treat.

[B50] Wenkel E, Thornton AF, Finkelstein D, Adams J, Lyons S, De La Monte S, Ojeman RG, Munzenrider JE (2000). Benign meningioma: partially resected, biopsied, and recurrent intracranial tumors treated with combined proton and photon radiotherapy. Int J Radiat Oncol Biol Phys.

[B51] Vernimmen FJ, Harris JK, Wilson JA, Melvill R, Smit BJ, Slabbert JP (2001). Stereotactic proton beam therapy of skull base meningiomas. Int J Radiat Oncol Biol Phys.

[B52] Weber DC, Lomax AJ, Rutz HP, Stadelmann O, Egger E, Timmermann B, Pedroni ES, Verwey J, Miralbell R, Goitein G, Swiss Proton Users Group (2004). Spot-scanning proton radiation therapy for recurrent, residual or untreated intracranial meningiomas. Radiother Oncol.

[B53] Noël G, Bollet MA, Calugaru V, Feuvret L, Haie-Meder C, Dhermain F, Ferrand R, Boisserie G, Beaudré A, Mazeron JJ, Habrand JL (2005). Functional outcome of patients with benign meningioma treated by 3D conformal irradiation with a combination of photons and protons. Int J Radiat Oncol Biol Phys.

[B54] Valentino V, Schinaia G, Raimondi AJ (1993). The results of radiosurgical management of 72 middle fossa meningiomas. Acta Neurochir (Wien).

[B55] Hudgins WR, Barker JL, Schwartz DE, Nichols TD (1996). Gamma Knife treatment of 100 consecutive meningiomas. Stereotact Funct Neurosurg.

[B56] Kurita H, Sasaki T, Kawamoto S, Taniguchi M, Terahara A, Tago M, Kirino T (1997). Role of radiosurgery in the management of cavernous sinus meningiomas. Acta Neurol Scand.

[B57] Chang SD, Adler JR, Martin DP (1998). LINAC radiosurgery for cavernous sinus meningiomas. Stereotact Funct Neurosurg.

[B58] Pan DH, Guo WY, Chang YC, Chung WY, Shiau CY, Wang LW, Wu SM (1998). The effectiveness and factors related to treatment results of gamma knife radiosurgery for meningiomas. Stereotact Funct Neurosurg.

[B59] Morita A, Coffey RJ, Foote RL, Schiff D, Gorman D (1999). Risk of injury to cranial nerves after gamma knife radiosurgery for skull base meningiomas: experience in 88 patients. J Neurosurg.

[B60] Shafron DH, Friedman WA, Buatti JM, Bova FJ, Mendenhall WM (1999). Linac radiosurgery for benign meningiomas. Int J Radiat Oncol Biol Phys.

[B61] Liscák R, Simonová G, Vymazal J, Janousková L, Vladyka V (1999). Gamma knife radiosurgery of meningiomas in the cavernous sinus region. Acta Neurochir (Wien).

[B62] Aichholzer M, Bertalanffy A, Dietrich W, Roessler K, Pfisterer W, Ungersboeck K, Heimberger K, Kitz K (2000). Gamma knife radiosurgery of skull base meningiomas. Acta Neurochir (Wien).

[B63] Roche PH, Régis J, Dufour H, Fournier HD, Delsanti C, Pellet W, Grisoli F, Peragut JC (2000). Gamma knife radiosurgery in the management of cavernous sinus meningiomas. J Neurosurg.

[B64] Villavicencio AT, Black PM, Shrieve DC, Fallon MP, Alexander E, Loeffler JS (2001). Linac radiosurgery for skull base meningiomas. Acta Neurochir (Wien).

[B65] Kobayashi T, Kida Y, Mori Y (2001). Long-term results of stereotactic gamma radiosurgery of meningiomas. Surg Neurol.

[B66] Shin M, Kurita H, Sasaki T, Kawamoto S, Tago M, Kawahara N, Morita A, Ueki K, Kirino T (2001). Analysis of treatment outcome after stereotactic radiosurgery for cavernous sinus meningiomas. J Neurosurg.

[B67] Stafford SL, Pollock BE, Foote RL, Link MJ, Gorman DA, Schomberg PJ, Leavitt JA (2001). Meningioma radiosurgery: tumor control, outcomes, and complications among 190 consecutive patients. Neurosurgery.

[B68] Spiegelmann R, Nissim O, Menhel J, Alezra D, Pfeffer MR (2002). Linear accelerator radiosurgery for meningiomas in and around the cavernous sinus. Neurosurgery.

[B69] Nicolato A, Foroni R, Alessandrini F, Maluta S, Bricolo A, Gerosa M (2002). The role of Gamma Knife radiosurgery in the management of cavernous sinus meningiomas. Int J Radiat Oncol Biol Phys.

[B70] Lee JY, Niranjan A, McInerney J, Kondziolka D, Flickinger JC, Lunsford LD (2002). Stereotactic radiosurgery providing long-term tumor control of cavernous sinus meningiomas. J Neurosurg.

[B71] Eustacchio S, Trummer M, Fuchs I, Schröttner O, Sutter B, Pendl G (2002). Preservation of cranial nerve function following Gamma Knife radiosurgery for benign skull base meningiomas: experience in 121 patients with follow-up of 5 to 9. Acta Neurochir Suppl.

[B72] DiBiase SJ, Kwok Y, Yovino S, Arena C, Naqvi S, Temple R, Regine WF, Amin P, Guo C, Chin LS (2004). Factors predicting local tumor control after gamma knife stereotactic radiosurgery for benign intracranial meningiomas. Int J Radiat Oncol Biol Phys.

[B73] Deinsberger R, Tidstrand J, Sabitzer H, Lanner G (2004). LINAC radiosurgery in skull base meningiomas. Minim Invasive Neurosurg.

[B74] Pollock BE, Stafford SL (2005). Results of stereotactic radiosurgery for patients with imaging defined cavernous sinus meningiomas. Int J Radiat Oncol Biol Phys.

[B75] Kreil W, Luggin J, Fuchs I, Weigl V, Eustacchio S, Papaefthymiou G (2005). Long term experience of gamma knife radiosurgery for benign skull base meningiomas. J Neurol Neurosurg Psychiatry.

[B76] Zachenhofer I, Wolfsberger S, Aichholzer M, Bertalanffy A, Roessler K, Kitz K, Knosp E (2006). Gamma-knife radiosurgery for cranial base meningiomas: experience of tumor control, clinical course, and morbidity in a follow-up of more than 8 years. Neurosurgery.

[B77] Kollová A, Liscák R, Novotný J, Vladyka V, Simonová G, Janousková L (2007). Gamma Knife surgery for benign meningioma. J Neurosurg.

[B78] Hasegawa T, Kida Y, Yoshimoto M, Koike J, Iizuka H, Ishii D (2007). Long-term outcomes of Gamma Knife surgery for cavernous sinus meningioma. J Neurosurg.

[B79] Feigl GC, Samii M, Horstmann GA (2007). Volumetric follow-up of meningiomas: a quantitative method to evaluate treatment outcome of gamma knife radiosurgery. Neurosurgery.

[B80] Davidson L, Fishback D, Russin JJ, Weiss MH, Yu C, Pagnini PG, Zelman V, Apuzzo ML, Giannotta SL (2007). Postoperative Gamma Knife surgery for benign meningiomas of the cranial base. Neurosurg Focus.

[B81] Iwai Y, Yamanaka K, Ikeda H (2008). Gamma Knife radiosurgery for skull base meningioma: long-term results of low-dose treatment. J Neurosurg.

[B82] Han JH, Kim DG, Chung HT, Park CK, Paek SH, Kim CY, Jung HW (2008). Gamma knife radiosurgery for skull base meningiomas: long-term radiologic and clinical outcome. Int J Radiat Oncol Biol Phys.

[B83] Takanashi M, Fukuoka S, Hojyo A, Sasaki T, Nakagawara J, Nakamura H (2009). Gamma knife radiosurgery for skull-base meningiomas. Prog Neurol Surg.

[B84] Ganz JC, Reda WA, Abdelkarim K (2009). Gamma Knife surgery of large meningiomas: early response to treatment. Acta Neurochir (Wien).

[B85] Collins SP, Coppa ND, Zhang Y, Collins BT, McRae DA, Jean WC (2006). CyberKnife radiosurgery in the treatment of complex skull base tumors: analysis of treatment planning parameters. Radiat Oncol.

[B86] Colombo F, Casentini L, Cavedon C, Scalchi P, Cora S, Francescon P (2009). Cyberknife radiosurgery for benign meningiomas: short-term results in 199 patients. Neurosurgery.

[B87] Yu JS, Yong WH, Wilson D, Black KL (2000). Glioblastoma induction after radiosurgery for meningioma. Lancet.

[B88] Kaido T, Hoshida T, Uranishi R, Akita N, Kotani A, Nishi N, Sakaki T (2001). Radiosurgery-induced brain tumor. Case report. J Neurosurg.

[B89] Shamisa A, Bance M, Nag S, Tator C, Wong S, Norén G, Guha A (2001). Glioblastoma multiforme occurring in a patient treated with Gamma Knife surgery. Case report and review of the literature. J Neurosurg.

[B90] Loeffler JS, Niemierko A, Chapman PH (2003). Second tumors after radiosurgery: tip of the iceberg or a bump in the road?. Neurosurgery.

[B91] Salvati M, Frati A, Russo N, Caroli E, Polli FM, Minniti G, Delfini R (2003). Radiation-induced gliomas: report of 10 cases and review of the literature. Surg Neurol.

[B92] Leber KA, Berglöff J, Pendl G (1998). Dose-response tolerance of the visual pathways and cranial nerves of the cavernous sinus to stereotactic radiosurgery. J Neurosurg.

[B93] Stafford SL, Pollock BE, Leavitt JA, Foote RL, Brown PD, Link MJ, Gorman DA, Schomberg PJ (2003). A study on the radiation tolerance of the optic nerves and chiasm after stereotactic radiosurgery. Int J Radiat Oncol Biol Phys.

